# “Will my fingerprint be enough?”: Secondary school students struggle to purchase a healthy, tasty and sustainable meal on the UK free school meal allowance

**DOI:** 10.1017/S1368980024002593

**Published:** 2025-01-08

**Authors:** Sundus Mahdi, Annie Connolly, Bob Doherty, Maria Bryant

**Affiliations:** 1 Department of Health Sciences, University of York, York, YO10 5DD, UK; 2 Trussell Trust, Unit 9, Ashfield Trading Estate, Ashfield Road, Salisbury, SP2 7HL, UK; 3 School for Business and Society, University of York, York, YO10 5DD, UK; 4 Hull York Medical School and the Department of Health Sciences, University of York, York, YO10 5DD, UK

## Supplementary Material

Supplementary Table 1

