# ‘Will my fingerprint be enough?’: secondary school students struggle to purchase a healthy, tasty and sustainable meal on the UK free school meal allowance

**DOI:** 10.1017/S1368980024002593

**Published:** 2025-01-08

**Authors:** Sundus Mahdi, Annie Connolly, Bob Doherty, Maria Bryant

**Affiliations:** 1 Department of Health Sciences, University of York, York, YO10 5DD, UK; 2 Trussell Trust, Unit 9, Ashfield Trading Estate, Ashfield Road, Salisbury, SP2 7HL, UK; 3 School for Business and Society, University of York, York, YO10 5DD, UK; 4 Hull York Medical School and the Department of Health Sciences, University of York, York, YO10 5DD, UK

**Keywords:** Free school meals, Food insecurity, Policy, Sustainability, School food, Citizen science, Co-production

## Abstract

**Objective:**

Free school meals (FSM) are a crucial form of support for families. This study aimed to investigate whether the FSM allowance can provide what is perceived to be, healthy, sustainable and satisfying food.

**Design:**

A mixed methods study incorporating co-production, citizen science and participatory approaches was conducted. Citizen scientists were given a daily budget equivalent to the FSM allowance and asked to purchase a ‘tasty, healthy and sustainable’ school lunch for a week. Alongside keeping records of available and purchased foods, young people engaged in focus groups to capture information on perceptions of food offered and FSM allowance adequacy.

**Setting:**

Secondary schools in Yorkshire, UK.

**Participants:**

Citizen scientists (*n* 42) aged 11–15 years across seven schools.

**Results:**

Obstacles were faced in obtaining sustainable and healthful meals when restricted to an FSM allowance. Reasons included restrictions in what could be purchased due to costs, limitations in the use of allowances that restricted breaktime purchases leading to hunger, inadequate portion sizes, systemic barriers like hurried lunch breaks that encourage ‘grab and go’ options and broken water fountains that led students to purchase bottled drinks. Findings were reinforced by descriptive food record data.

**Conclusions:**

Our findings suggest that schools would benefit from national policies to address the lack of funding, infrastructure issues and capacity to support optimal provision of food to those on FSM as well as provide greater flexibility in how pupils use their allowance. Young people verified these findings, which they presented to policymakers at a parliamentary event.

Since the UK cost-of-living crisis in late 2021, the number of families and children living in poverty has risen. The cost-of-living crisis was when the cost of essential goods had increased due to inflation whilst household incomes remained static. Food insecurity has been found to impact 20–25 % of households with children^(1)^, leading to insufficient food, inadequate diets^(2)^ and negative health impacts on weight status^(3)^. Both maintained (i.e. state) schools and academies (i.e. government-funded independent schools) have an obligation to offer means-tested free school meals (FSM) to pupils between the ages of 5–16 in England, whose families have an annual household income of < £7400 before benefits^(4)^. Based on these criteria, almost 25 % of UK children are eligible for FSM^(5)^.

At the time of writing, the FSM daily allowance rate was £2·53 in secondary schools, set by the UK government through the National Funding Formula. This funding is transferred to local authorities or multi-academy trusts, based on the number of FSM registrations. Through a School Forum, a consultative body comprising representatives from schools and academies, an FSM allowance rate is agreed upon locally. This may differ by local authority and may be more or less than the funding allocated by the government. Funding is then transferred to schools/multi-academy trusts based on FSM registrations. Each school offers a set meal each day priced at the FSM allowance for the school^(6)^. As such, schools may choose to provide a different FSM allowance than the original £2·53. Pupils often have a choice of foods to purchase at lunchtime, whether as single items or set meals, or they can opt for a packed lunch. Although those on FSM have a daily allowance, all pupils can purchase more than the FSM allowance, should they have the funds to do so. There is no nutritional requirement of what can be purchased, though all schools must provide nutritional options based on school food standards^(7)^. Payments are usually through a cashless system, such as a unique student payment card or an online account.

The provision of FSM to eligible pupils in England is a long-standing policy aimed at addressing socio-economic inequalities. Evidence indicates an effect on academic performance^(8,9)^, attendance^(10)^ and reduced behavioural problems^(11)^. In addition, FSM have an ability to reduce socio-economic inequalities in diet^(12)^, can improve overall dietary quality and have been found to be healthier than packed lunches^(10,13)^.

There is currently limited research investigating the relationship between food prices within school canteens and young people’s choices. One study has explored the impact of price differences in healthy and unhealthy foods in secondary schools in Scotland and found that it disadvantaged those from lower socio-economic backgrounds, restricts affordability and leads to health inequalities regarding access to nutritious meals^(14)^. In other research, discussions with parents suggested concerns regarding the healthiness of foods on offer, the affordability of healthy options and the ability of portion sizes served on the FSM allowance to satisfy children’s hunger, calling for an increase to the FSM allowance^(15)^.

Given the increasing food costs driving inflation, there is also more need than ever to investigate the extent to which the FSM allowance enables or restricts students’ ability to choose healthy food at school. This study aimed to use a participatory approach to (1) investigate whether the allowance was able to provide what young people perceive to be healthy, sustainable and tasty food and (2) investigate the types of food available to those in receipt of FSM in English secondary schools. The research topic was agreed upon in consultation with secondary school pupils through the Food Foundation’s Young Food Ambassadors.

## Methods

### Design

**Figure 1.**
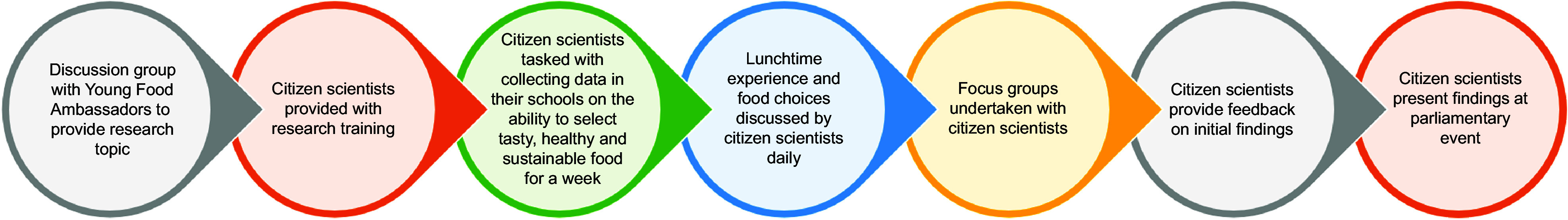
Study flow of activities.

A mixed methods study was conducted incorporating a citizen science approach and was underpinned by meaningful involvement with young people, including participatory and co-production approaches (see Fig. 1). Co-production is the involvement of the research population in the design, delivery and dissemination of the research^(16)^. Throughout the manuscript, young people will be referred to as ‘citizen scientists (CS)’.

### Recruitment

Six secondary school pupils, aged 11–15 years (years 7–10), from each of seven Yorkshire (UK) schools (*n* 42) were invited to take part in the project that involved citizen science, research participation and co-production to capture information on, and perceptions of, the food available at their schools. Invitation to take part came via an approach and nomination by their respective schools through purposive and convenient means. Schools were recruited through previously established relationships from previous partnerships and collaborations, as part of the wider FixOurFood in Schools project^(17)^. Students were not asked to disclose their FSM eligibility for ethical reasons. To increase the likely understanding of FSM by CS and to increase the likelihood of CS being on FSM, schools had to have over the national average of FSM eligibility of 24 %. Schools were additionally sampled to get a broad range of characteristics, including variability in catering provisions.

### Training day

The research team hosted an interactive training day in March 2023, whereby recruited CS were provided with training and skills to collect data in their schools. A co-produced group charter of values and principles was co-authored by all attendees, and expectations were set for the duration of the project. Basic research methods, project aims and methods were outlined. In addition, research terminology and principles were also explained through group discussions and examples^(18)^, facilitated by topic experts. Young researchers were also asked to consider concepts of ‘healthy’ and ‘sustainability’ from their own perspectives. CS were briefed on how to record data in the research folder, alongside the provision of and instructions on how to use a Dictaphone. A co-production approach was utilised where young people were consulted on how best to undertake the research within their schools. Ongoing support on how to conduct the research and collate data was provided to CS after the training day. For instance, CS were told that a researcher was available to be contacted during the research week to discuss any issues. Our citizen science approach actively involved young people from research conception to dissemination. For example, methods used to gather data and avoid barriers were developed during training by the young people whilst researchers worked closely with CS to develop dissemination approaches.

### Data collection

Participating schools received the equivalent of 1 week’s FSM allowance to cover the costs of CS’ meals, irrespective if they were currently in receipt of FSM or not. Payment of meals by CS was through the same processes as other pupils, including those on means-tested FSM, in their respective schools (though this may vary between schools). Most schools used thumbprint scanners, which were linked to their accounts.

CS were provided with a research folder and, over 1 week, were asked to record their food purchases over the school day, the prices of food and the availability and content of meal deals (meals usually set at the level of the FSM allowance). Food records were not intended for dietary assessment, nor were they intended to capture a comprehensive record of foods consumed. Rather, collecting food records helped provide insights into popular foods consumed. Further records were made on whether their chosen food contained fruit or vegetables, whether they would have made the same choices if they had a larger allowance and to what extent the food had filled them up on a 4-point Likert scale (1 – yes, fully full; 2 – somewhat full; 3 – not very full; 4 – still very hungry). In addition, CS were encouraged to meet for daily audio-recorded group discussions to discuss what they had bought that day, using the Dictaphones provided. Research activities took place at the end of lunchtime or by the end of each day at the latest.

Within 4 weeks after the completion of the FSM allowance research week, CS took part in focus groups (45–60 min) to discuss their perceptions of the food on offer whilst they were restricted to the FSM allowance (see Table 1 for an overview of the topic guide). Seven focus groups were conducted in total.

**Table 1.** Focus group topic guide overview

(1) Opinions on whether the food/drinks purchased were tasty, healthy and sustainable
(2) Feedback on whether the food was filling
(3) Insights into food wastage
(4) What pupils were and were not able to buy with their allowance throughout the school day
(5) Whether pupils would choose a different meal if they had more money to spend
(6) What would be considered a suitable allowance to enable pupils to buy a tasty, healthy and sustainable meal.

A member of the research team (AC) had a school dinner in every school with the CS and was able to gather observations about the dining environment and food on offer to help facilitate focus group discussions and interpretation of data (results not reported).

### Data analysis

Focus groups and post-lunch discussions between CS were recorded, transcribed and analysed using thematic analysis by two researchers (AC and SM)^(19)^. A grounded theory inductive approach was undertaken, whereby evidence was coded thematically and grouped into themes. Any discrepancies in coding were discussed between researchers until a consensus was reached. In addition, an explorative and descriptive analysis of the food records was undertaken (SM and AC). Frequencies were calculated to describe CS food choices and food preferences daily (see online supplementary material, Supplemental Table 1) and as a total of the whole week. Each food item was treated independently of the others (i.e. choice of food items is not mutually exclusive), and therefore, a summed frequency across food items will not equal the total number of CS. In addition, not all food items consumed over lunch have been listed and reported.

## Results

All forty-two CS attended the research training day; thirty-eight completed food records throughout the week, thirty-five attended focus groups and all contributed to data interpretation, co-production of the report and dissemination activities. The FSM allowance provided varied by school, ranging from £2·15 to £2·70, as did the meals offered alongside the lunchtime experience. The themes developed as a result of the focus groups’ reflection on the research week fell into two broad categories: allowance use or wider school food system factors. These themes have been presented in accordance with primary (directly aligned to the research question) and secondary findings (other emergent themes).

### Primary findings

#### Allowance use

##### Allowance adequacy and restrictions in use

Findings suggested that the current FSM allowance was inadequate. The limited funds available meant that the allowance was not sufficient to cover food costs for both break and lunch.

*So if you were on free school meals and you went up at break they would just turn you away, they wouldn’t even consider giving you anything, that’d be it you were done with. (S7)*

It was common across most schools that CS could not access their FSM allowance during the morning break, which often led them to go hungry up until lunchtime, considerably so for those who had not had breakfast.

*We can’t get snacks at break time that, kind of, like doesn’t take me till lunch. So I get hungry like during the like morning. (S3)*

*If you want to get something at lunch, you can’t get anything at break, which means that you have to go from when you have your breakfast at like seven o’clock till one o’clock. (S4)*

##### Restricted choices with allowance (meal deals and variety of options)

Meal deals offered a mains, a dessert and sometimes a drink or a choice between a dessert or drink. It was highlighted that the meal deal offered the best value when restricted to an FSM allowance; this meant reduced choice, however. For instance, fruit pots were unaffordable alongside a meal deal option and rarely were they offered as part of the meal deal.

*You wouldn’t be able to get it [fruit pots] with like a full, full meal like with water and like a full meal as well. Like, some of the food is take up a lot of your budget. (S3)*

It was expressed that the current FSM allowance limited the choice and variety of options that they could choose from at lunch, including having repetitive meals on a weekly basis.

*Interviewer (I): Has anybody got any comments about whether you think you’ve got a good choice with your £2.35? Participant (P): If we have more variety it makes sense. But we don’t have, we have less variety and less portion, less everything to be exact. (S2)*

*… I think you are very limited on what you can buy because most things are pretty expensive. (S3)*

The lack of variety was also said to impact their ability to make healthy choices, such as the variety of fruit within fruit pots or the variety of vegetables within salads.

*And mostly full of lettuce and the other option like tomato, cucumber, not enough of that. (S6)*

##### Cost barriers to healthy choices

CS from most schools believed that food options on sale were overpriced and unaffordable, especially for the portion sizes served, deeming the allowance inadequate to purchase a filling meal. Those on FSM were unable to spend more than their allowance (i.e. go into debt). CS highlighted the increasing costs of food prices whilst the FSM allowance remained unchanged.

*But it used to be that in Year 7, 8 and 9… you could get like a bottle of water or a carton of something and a home bake and like a main all for within that budget and now I barely get a slice of pizza, which is only about that big and they’re like, ‘Oh, sorry, all you can afford now is a bottle of water’. (S7)*

It was also highlighted that healthier food was more expensive and that school food prices had gone up, in comparison with the allowance received, and therefore, pupils were getting less for their money. Suggestions for cheaper-priced healthy meals and fruit were made to increase uptake.

*It’s more the allowance is alright I think, it’s just the amount that everything’s been priced at rather than the actual money that you’ve got. (S7)*

CS additionally highlighted that fruit was rarely sold during break and that the food was unhealthy and overpriced, making it difficult to afford.

*But with the things at break, it’s definitely over-priced. You get a small, incredibly greasy pizza for like £1.10. (S1)*

##### Suggested recommendations to allowance and usage

There was a popular opinion that the FSM allowance needed to be increased to be able to purchase a meal that would fill young people up for longer. In those schools where a drink was not provided, access to a beverage through an increased allowance was a popular opinion. Others voiced that an increased allowance would enable them to afford food during both break and lunchtime. There was also a call for flexibility on how the allowance can be spent. For instance, it was suggested that any underspent allowance should be rolled over and that pupils should be able to spend over their allowance on one day, with it being deducted from the following day’s allocation. Others voiced that an increased allowance would provide them with more options on what food items they can buy, including making it easier to buy healthier and more sustainable alternatives, and more food items if needed.

*Even if they changed it [the allowance] to maybe you get money in your account weekly because then you’ll get more choice… like if you’re more hungry on another day than you are other, or if you have PE where you’d 100 % want a water bottle… (S1)*

#### Wider school food system factors

##### Importance of menus and clear pricing

In many schools, food items were not priced clearly, or at all, and menus could not be seen in advance. It was also highlighted that prices were only accessible at either point of purchase (e.g. when checking out) or when choosing food items (e.g. a wall with all the prices indicated). Others highlighted that pricing was only available for meal deal items This meant CS restricted to the FSM allowance often had to make choices quickly whilst in the queue. Having to choose under pressure sometimes made it more difficult to choose the healthiest options.

*You’ve still got to look, guess and pay and that’s how it goes, you’ve got no more time to think would that fill me?… It’s sort of just I see that, I’m going to have it, it’s in my budget. (S7)*

*When you get to the front it’s like you pick your stuff and you go because people behind you are just like so impatient…(S7)*

In some instances, not having clear prices led to feelings of embarrassment, particularly where the FSM allowance was not sufficient to cover the cost of the chosen meal and CS had to return food items.

*I felt embarrassed because I was like, ‘Oh…’… because there are other people that are waiting in line right behind you, like I’m here to get my food that I can afford. (S4)*

The lack of access to the school lunch menus beforehand, alongside clear pricing, was deemed important given funding restrictions with the FSM allowance. To overcome this, CS reported that pupils had to try and remember what was normally served on that day or look before queuing. In instances where what was being served was different to what pupils were routinely accustomed to led to confusion.

*If you wanna like know what’s on, you have to like go like in between, not in the line, but have a look like that. (S1)*

##### Availability of healthy and sustainable food

It was a dominant opinion that most school food offered was not healthy.

*… Mainly pasta because it’s just one of the things that’s… it’s not the nicest and it’s not the healthiest but it’s one of the most filling and less expensive. (S7)*

CS faced restrictions to their ability to choose healthy items. For instance, pizzas were often served without any vegetables or a salad option, there was a limit on fruit options served and, in some cases, no fruit options were available.

*The closest thing you get to fruit is jelly. (S7)*

There was additionally a lack of vegetables offered in or within meals, and in one school, this meant no vegetarian options.

*. … couldn’t get anything vegetarian, and it was like difficult for them to find things for and they couldn’t afford to keep buying packed lunches. (S7)*

Despite these shortcomings, CS from two schools praised the availability of free fruit and vegetables within school meals, indicating the availability of free fruit and salad, whilst another indicated that they had access to an unlimited salad when it was available.

*There is a salad which is optional, which is free… the salad isn’t always there as well, it’s just a little bit. (S6)*

##### Availability of high-quality and tasty food

Some CS expressed their concerns regarding the quality and taste of school meals. This included complaints about the texture of food, which was flagged as either oily and greasy, stale or dry. Others complained that the food was uncooked, not fresh and generally low quality (both in how the food is prepared and in appearance). Some felt that the food offered was not tasty, particularly healthier and more sustainable/vegetarian meal options.

*I think the food wasn’t extremely healthy. The pizza was not fresh, I would say it was frozen. And then the chips, they were kind of hard, kind of soggy as well. (S2)*

#### Secondary findings

##### Water and drinks

CS highlighted issues with accessing water during the school day. Although most schools had water fountains, they were reportedly often broken. Others highlighted that there were a limited number of water fountains around the school which made refilling water bottles difficult.

*It’s always broken, to be honest. So some students can’t get more water. (S2)*

Many CS did not trust the use of water fountains due to hygiene concerns, claiming the water to be unclean.

*There’s a water fountain but it’s often broken and sometimes the water comes out gross. (S7)*

In the absence of functioning and hygienic water fountains, CS had no choice but to purchase water as part of their meal deal. It was flagged that water bottles on sale separate from meal deals were small and expensive.

##### Lunchtime system and food access

The limited time for a lunchbreak was highlighted by many of the CS as a key issue. Long queuing times and short lunch breaks (ranging from 25 to 35 min) meant that pupils had limited time to eat, which resulted in unhealthy choices, such as ‘grab and go’ options (e.g. pizza on a napkin). The sandwiches or pizza were chosen over a full hot meal, which was often the healthier option, as this took longer to queue for and eat. The limited time to eat also led to food wastage.

*Some people…they throw out the food because they don’t have enough time to eat it all. (S2)*

##### Sustainable packaging and food waste

Nearly every pupil got a drink in a plastic bottle with their lunch (either as part of a meal deal or purchased separately). Not only did this lead to the consumption of sugary drinks in some cases, but it also meant that schools often had a high level of plastic usage and disposal. In one school, it was reported that this equated to thousands of bottles a week.

*We did the maths… we got the result of 55,000 something like that, a year so that’s probably like doubled per year for one school. (S7)*

The use of plastics was also common in cutlery, the wrapping of cold foods and pre-packaged food items such as desserts. Most schools did not serve hot food on plates, but rather in a box.

*We never had plates… we used to have actual knives and forks… You have plastic knives and forks now… (S2)*

Recycling was flagged as a concern in four schools. In some cases, this meant no access to recycling bins despite the use of recyclable cutlery.

*The bad thing about this school is we don’t recycle. (S7)*

Some schools used more sustainable options. These included paper-based packaging as opposed to plastic packaging, washable plates and cutlery, availability of recycling bins and serving water in glasses.

*Where they had a dinner lady at break time who had glasses of water and she’d fill it up and you could have a glass of water. (S2)*

#### Food records

CS daily food choices were explored and summed over the week (*n* 190 food records), alongside their perceptions of the foods selected (see Table 2). Approximately 21 % of 158 food records included pizza; 22 % included chips, fries or potato wedges; and 47 % were bread-based meals, such as paninis and sandwiches. For dessert, 8 % of 118 records specified a fruit option, whereas 84 % specified a sugar-based option (e.g. cakes and biscuits). In two out of the five research days, no fruit options were selected by any of the CS. There was an almost equal selection of water and other drinks purchased across the week. Across 134 food records, there was mixed feedback regarding how filling purchased meals were; 44 % of food records reported to be ‘fully full’, and just over 20 % of food records reported not being fully satisfied. Based on 125 food records, 42 % of meals did not include any fruit or vegetables based on CS reports. When asked whether they would buy the same meal if they had more money, CS responded ‘no’ in 43 % of ninety-five cases. When asked whether they would have chosen a different meal if they had not been tasked with choosing ‘tasty, healthy and sustainable food’, the response was ‘no’ in 57 % of eighty-one cases.

**Table 2.** Summary of citizen scientists’ (*n* 38) food choices and perceptions summed across the week (*n* 190)

Item	Total across all week (*n* 190)*	% of sample across week†
Mains		*n* 158
Pizza	33	20·89
Bread‡	75	47·47
Pasta	19	12·03
Chips§	34	21·52
Other	23	14·56
Missing	32	–
Dessert		*n* 118
Fruit	9	7·63
Sugar-based||	99	83·90
None	10	8·47
Missing	72	–
Drink		*n* 121
Water	54	44·63
Other¶	57	47·11
None	10	8·26
Missing	69	–
Were fruit and vegetables present in meal?		*n* 125
No	52	41·60
Yes	73	58·40
Missing	65	–
If you had more money, would you have chosen the same thing?		*n* 95
Yes	54	56·84
No	41	43·16
Missing	95	–
If you weren’t selecting ‘tasty, healthy, sustainable food’, would you have chosen something else?		*n* 81
Yes	35	43·21
No	46	56·79
Missing	109	–
Did the food you were able to buy at lunchtime fill you up?		*n* 134
Fully full	59	44·03
Somewhat full	45	33·58
Not very full	20	14·93
Still very hungry	10	7·46
Missing	56	–

* The number of food items consumed within ‘mains’ is independent of the number of food records; therefore, the column total will exceed the 190 food records. For instance, pupils could consume both pizza and chips. The number of missing data refers to the number of pupils who did not complete a food record.

† Percentage of reported data based on the number of food records across the week. This has been calculated as the total of 190 entries minus the sum of missing data over 5 d. Columns do not equal 100 % within ‘mains’ as food items are not mutually exclusive and have been analysed independently, unlike other items reported in the table.

‡ Meals that included paninis, baguettes, garlic bread, wraps, naan, loaf and burgers.

§ This included chips, fries and wedges.

|| This included cakes, biscuits, baked goods and other desserts containing refined sugar.

¶ This included fruit juice, Radnor Fizz and other flavoured water.

## Discussion

This study aimed to investigate, using participatory and citizen science approaches, whether the FSM allowance was sufficient for secondary school pupils to buy what they perceived to be healthy, tasty and sustainable meals. Several barriers to achieving this goal were identified, primarily involving limitations in allowance use and issues with the school food system.

*Allowance use* explored how the FSM allowance was and could be used and what options were available to CS. Study findings highlighted that the meal deal offered the best value for money and was often the only option available within the FSM allowance, therefore limiting food choices and the ability to afford healthier alternatives, which were usually more expensive. This was particularly a problem as portion sizes were often deemed inadequate to sustain pupils throughout the school day. Small portion sizes have been flagged in previous research where the FSM allowance was deemed insufficient to provide a well-portioned meal leading to parents subsidising costs^(15)^.

Barriers within the *school food system* meant that some schools lacked the availability of tasty, healthy and sustainable food choices. This was reflected within CS food records, which showed that very little fruit and vegetables were purchased. Existing research has continued to highlight the lack of quality of school meals, and that the consumption of ultra-processed foods is highest in those from the lowest income^(13)^. Existing research has also reported negative student perceptions of healthy food served at school, where fruit was deemed low quality and the availability of healthy options was scarce^(20)^. This is further verified in a survey whereby 55 % of 324 families and children said their school offered vegetables every day, whilst 13 % said their school did not offer vegetables at all^(14)^. Despite this, recent evidence has suggested that schools do not view school food standards as a priority^(21)^, despite being mandatory. This highlights the importance of making improvements to the school food offer; accessing a nutritious meal can be further constrained by the limited choices available with a restricted allowance.

It was a popular opinion that school food was expensive and food prices were rising whilst the allowance was not. Over 40 % of young people in our study reported that they would have purchased a different meal if they had a greater allowance. Increasing food prices in the UK have impacted school meal quality and costs, particularly for healthier options^(22–24)^. Soaring school food costs has meant that 75 % of secondary school pupils regularly purchase lunch outside of school, seeking food that is better value for money^(20)^.

Pupils’ use of governmental support such as the FSM allowance is limited by the current school offer and policies which could further widen health inequalities. Many of the CS were unable to use their allowance during the breaktime, leading some to feel hungry all morning. There were also feelings of stigma with being unable to purchase a breaktime snack like those not receiving FSM. It has been previously reported that almost 60 % of pupils consume a snack during the morning break^(20)^ and that feelings of shame and difference are experienced by pupils unable to afford food from school^(25)^. Moreover, almost 25 % of UK secondary school pupils from lower socio-economic backgrounds or attending schools with high FSM registrations (20–40 %) do not consume breakfast, in comparison with 6 % from higher socio-economic backgrounds^(20)^. This would suggest that a greater proportion of pupils who are on FSM are not consuming any food until lunchtime due to FSM allowance use restrictions during the morning break. Previous research has highlighted the advantages of consuming food in the morning as an obesity prevention measure^(26)^ and the negative impacts of hunger on school attainment^(27)^.

We found that inequalities in the *wider school food system* for those receiving FSM were detrimental to the lunchtime experience and the ability to opt for healthier and more sustainable diets. For instance, any underspent allowance could not be rolled over or accumulated, unlike those not receiving FSM who were able to use their account balance as and when desired. The inability to go over the daily allowance led to instances where pupils had to return food items leading to embarrassment. Limited access to menus and a lack of clear pricing meant that pupils had to make rushed food choices at the counter, which were often not the healthiest.

CS reported the lack of access to clean water due to faulty water fountains and hygiene concerns, leading to increased purchases of bottled drinks with a limited allowance. Long queuing times and little time to eat also meant that pupils had to opt for quick and often unhealthier ‘grab and go’ options rather than a full hot meal. These findings have been demonstrated within previous research on the barriers to making healthy food choices in UK secondary schools^(15,28)^. Although schools ought to consider increasing the availability and affordability of healthier options to encourage healthy and sustainable food choices, they are often unable to meet current demands given conflicting priorities, funding cuts and high inflation rates, which often means that school lunches are overlooked^(29,30)^.

Taking a participatory approach led to high levels of engagement with the project. CS had the opportunity to take part in the dissemination of research findings. Online discussion sessions were undertaken with each school to gather CS comments, to verify that the data had been correctly interpreted and that they agreed with the proposed policy recommendations. Since this research has been conducted, CS have taken steps within their own schools and have since worked with the caterers and school leadership to tackle some of the issues outlined in this research, including the introduction of clear pricing on all food items. CS were also involved in the production of a report (via the discussion sessions) and were involved in planning and designing a UK parliamentary event in November 2023, where they presented findings and national policy recommendations at the House of Lords to policymakers. These are consistent with those advocated elsewhere^(31,32)^ and include (1) ring-fencing the FSM allowance so that pupils are receiving the daily £2·53 allocation provided by the central government, (2) greater flexibility in how pupils could use their allowance allowing for any rollover or accumulation of any underspend, (3) introducing a requirement within school food standards to offer at least two portions of vegetables with every meal and (4) ensuring schools have sufficient funding to improve their lunchtime infrastructure including maintenance of water fountains and recycling of packaged items^(6)^. Alongside the report, several of the CS wrote and presented poems highlighting the issues experienced by students receiving FSM^(33)^ and discussed research findings with UK Members of Parliament (elected officials who serve in the House of Commons) and members of the House of Lords^(34)^.

Taking a participatory approach has also improved our understanding of appropriate methods to use when undertaking studies in this area. Although food records were not intended for dietary assessment analysis, they were useful in collecting observational and descriptive data and provided a context for focus groups. However, CS fed back that the food records were too long and repetitive; this led to high levels of missing, and difficult to interpret, data. They also shared that they didn’t find daily group discussions comfortable or easy to organise. Although we have reported what was disclosed in food records, our conversations during the focus groups suggested a variance in the definition of foods and food groups on occasion (e.g. tomato sauce was defined as a vegetable). Similarly, focus group discussions suggested a variance in interpretation or understanding of the terms ‘sustainable’ and ‘healthy’, which could have impacted food choices and their responses. Future studies would benefit from co-producing and piloting outcome measures with citizen scientists to maximise data quality. In addition, this research specifically tasked pupils to choose healthy and sustainable meals, which may not be reflective of actual behaviour. Although in most instances food choices did not reflect healthy and sustainable choices, this may have been due to limitations in the school food offer. Additionally, almost 40 % of food records reported that they may have chosen a different meal if they were not participating in this research. This may be due to several reasons, such as not being restricted to the FSM allowance. The collection of empirical data is warranted within future research, such as the associations between food expenditure and food choice throughout the school day. Finally, due to the nature of this study being a citizen science project, we were not seeking transferability of findings. Therefore, additional descriptive information from schools was not collated and compared (e.g. school participation in health-promoting schemes or school food adherence to school food standards) that could have helped provide a context to our research findings. As such, the experiences of citizen scientists may not be generalisable to other schools.

### Conclusions

The present study has highlighted concerning barriers to secondary school pupils accessing healthy and sustainable meals, most likely linked to the limited FSM allowance, flexibility in how it can be used, school infrastructure and lunchtime systems and the soaring costs of food prices and inflation that has negatively impacted school funding streams. This has led to several recommendations set out by the young people, including amendments to school food standards and increasing funding to schools to support improvements to school food provision. These recommendations are aligned to those advocated elsewhere and warrant consideration.

## Supporting information

Mahdi et al. supplementary material Mahdi et al. supplementary material

## Supplementary material

For supplementary material accompanying this paper, visit https://doi.org/10.1017/S1368980024002593.

## References

[1] The Food Foundation Food Insecurity Tracking. https://foodfoundation.org.uk/initiatives/food-insecurity-tracking#tabs/Round-14 (accessed October 2024).

[2] Shi Y , Davies A & Allman-Farinelli M (2021) The association between food insecurity and dietary outcomes in university students: a systematic review. J Acad Nutr Diet 121, 2475–2500. doi: 10.1016/j.jand.2021.07.015. 34352437

[3] Morales ME & Berkowitz SA (2016) The relationship between food insecurity, dietary patterns, and obesity. Curr Nutr Rep 5, 54–60. doi: 10.1007/s13668-016-0153-y. 29955440 PMC6019322

[4] Department for Education (2018) Free School Meals: Guidance for Schools and Local Authorities. https://www.gov.uk/government/publications/free-school-meals-guidance-for-schools-and-local-authorities (accessed October 2024).

[5] Department for Education (2024) Academic Year 2023/24: Schools, Pupils and their Characteristics. https://explore-education-statistics.service.gov.uk/find-statistics/school-pupils-and-their-characteristics (accessed October 2024).

[6] Connolly A , Bryant M , Brinsden H et al. (2023) A Better Deal for Free School Meals. The Food Foundation. https://foodfoundation.org.uk/sites/default/files/2023-11/TFF_FSM%20Allowance_Report_FINAL.pdf (accessed October 2024).

[7] Department for Education (2024) Guidance: School Food Standards Practical Guide. https://www.gov.uk/government/publications/school-food-standards-resources-for-schools/school-food-standards-practical-guide (accessed October 2024).

[8] CDC Healthy Schools (2021) Making the Connection: Dietary Behaviors and Academic Grades. https://www.cdc.gov/healthyschools/health_and_academics/health_academics_dietary.htm (accessed October 2024).

[9] de Oliveira KHD , de Almeida GM , Gubert MB et al. (2020) Household food insecurity and early childhood development: systematic review and meta-analysis. Matern Child Nutr 16, e12967. doi: 10.1111/mcn.12967. 32052571 PMC7296813

[10] Cohen JF , Hecht AA , McLoughlin GM et al. (2021) Universal school meals and associations with student participation, attendance, academic performance, diet quality, food security, and body mass index: a systematic review. Nutrients 13, 911. doi: 10.3390/nu13030911. 33799780 PMC8000006

[11] Shankar P , Chung R & Frank DA (2017) Association of food insecurity with children’s behavioral, emotional, and academic outcomes: a systematic review. J Dev Behav Pediatr 38, 135–150. doi: 10.1097/DBP.0000000000000383. 28134627

[12] Petrovic D , de Mestral C , Bochud M et al. (2018) The contribution of health behaviors to socioeconomic inequalities in health: a systematic review. Prev Med 113, 15–31. doi: 10.1016/j.ypmed.2018.05.003. 29752959

[13] Parnham JC , Chang K , Rauber F et al. (2022) The ultra-processed food content of school meals and packed lunches in the United Kingdom. Nutrients 14, 2961. doi: 10.3390/nu14142961. 35889918 PMC9318725

[14] Kenton-Lake S , Heger J , Tobi R et al. (2021) Feeding Our Future: An inVEGtigation Into UK School Food 2021. The Food Foundation. https://foodfoundation.org.uk/sites/default/files/2021-10/Peas-Please-School-Food-Report-2021-Spreads.pdf (accessed October 2024).

[15] Sahota P , Woodward J , Molinari R et al. (2014) Factors influencing take-up of free school meals in primary- and secondary-school children in England. Public Health Nutr 17, 1271–1279. doi: 10.1017/S136898001300092X. 23578731 PMC10282406

[16] Smith H , Budworth L , Grindey C et al. (2022) Co-production practice and future research priorities in United Kingdom-funded applied health research: a scoping review. Health Res Policy Syst 20, 36. doi: 10.1186/s12961-022-00838-x. 35366898 PMC8976994

[17] FixOurFood Sustainable and Healthy Food for Children. https://fixourfood.org/what-we-do/our-activities/schools-and-nurseries/ (accessed October 2024).

[18] School Food Plan (2015) The School Food Standards. https://www.schoolfoodplan.com/wp-content/uploads/2015/05/School_Food_Standards_140911-V2e-tea-towel.pdf (accessed October 2024).

[19] Braun V & Clarke V (2006) Using thematic analysis in psychology. Qual Res Psychol 3, 77–101. doi: 10.1191/1478088706qp063oa.

[20] Wills W , Danesi G , Kapetanaki AB et al. (2019) Socio-economic factors, the food environment and lunchtime food purchasing by young people at secondary school. Int J Environ Res Public Health 16, 1605. doi: 10.3390/ijerph16091605. 31071922 PMC6540591

[21] Kantar Public (2023) School Food Standards Compliance Pilot: Discovery and Feasibility Research. Food Standards Agency, Kantar Public, Department for Education. https://www.food.gov.uk/research/innovative-regulator/school-food-standards-compliance-pilot-discovery-and-feasibility-research (accessed October 2024).

[22] Goudie S & Hughes I (2022) The Broken Plate 2022: The State of the Nation’s Food System. The Food Foundation. https://foodfoundation.org.uk/sites/default/files/2022-07/The%20Broken%20Plate%202022%20report.pdf (accessed October 2024).

[23] English A & Tobi R (2023) Food Prices and Profits during the Cost-of-Living Crisis. The Food Foundation. https://foodfoundation.org.uk/sites/default/files/2023-09/TFF_PROFIT%20BRIEFING_Final.pdf (accessed October 2024).

[24] Adams R (2023) ‘Cost of Eating’ Crisis: Price of School Lunches Up by a Third in Parts of England. The Guardian. https://www.theguardian.com/education/2023/may/01/cost-of-eating-crisis-price-of-school-lunches-rise-by-third-in-parts-of-england-since-2019 (accessed October 2024).

[25] Carlisle VR , Jessiman PE , Breheny K et al. (2023) A mixed methods, quasi-experimental evaluation exploring the impact of a secondary school universal free school meals intervention pilot. Int J Environ Res Public Health 20, 5216. doi: 10.3390/ijerph20065216. 36982124 PMC10049258

[26] Mahdi S , Dickerson A , Solar GI et al. (2023) Timing of energy intake and BMI in children: differential impacts by age and sex. Br J Nutr 130, 71–82. doi: 10.1017/S0007114522003014. 36128754 PMC7614627

[27] Littlecott HJ , Moore GF , Moore L et al. (2016) Association between breakfast consumption and educational outcomes in 9–11-year-old children. Public Health Nutr 19, 1575–1582. doi: 10.1017/S1368980015002669. 26411331 PMC4873891

[28] McSweeney L , Bradley J , Adamson AJ et al. (2019) The ‘Voice’ of key stakeholders in a school food and drink intervention in two secondary schools in NE England: findings from a feasibility study. Nutrients 11, 2746. doi: 10.3390/nu11112746. 31726753 PMC6893517

[29] National Education Union (2023) Current School Funding Levels are Inadequate. https://neu.org.uk/press-releases/current-school-funding-levels-are-inadequate (accessed October 2024).

[30] Hacking V (2022) APPG on School Food Report: Impact of Food Cost on School Meals. https://apse.org.uk/index.cfm/_api/render/file/?fileID=C0ED6D45-71C0-496F-B090D83C42BE1F85 (accessed October 2024).

[31] Schliemann D , Spence S , O’Kane N et al. (2024) Identifying the top 10 research priorities for the school food system in the UK: a priority setting exercise. BMJ Open 14, e081400. doi: 10.1136/bmjopen-2023-081400. PMC10941135 38485482

[32] School Food Review Working Group (2023) Statement on Funding. https://www.schoolfoodmatters.org/sites/default/files/2022-10/Statement%20on%20School%20Food%20Funding%20.pdf (accessed October 2024).

[33] The Food Foundation (2023) School Students Campaign for Better School Meals in Parliament. https://foodfoundation.org.uk/news/school-students-campaign-better-school-meals-parliament (accessed October 2024).

[34] UK Parliament House of Lords. https://www.parliament.uk/business/lords/ (accessed October 2024).

