# Supplementary material for: ‘Will my fingerprint be enough?’: secondary school students struggle to purchase a healthy, tasty and sustainable meal on the UK free school meal allowance
Source: Public Health Nutr. 2025 Jan 8;28(1):e19. doi: 10.1017/S1368980024002593 (PMC7617354; doi:10.1017/S1368980024002593)
Supplement: Mahdi et al. supplementary material [file S1368980024002593sup001.docx]

| **Supplementary Table 1.** Frequency of citizen scientists’ food choices and perceptions daily (n=38) | | | | | |
| --- | --- | --- | --- | --- | --- |
| **Item** | **Day 1** | **Day 2** | **Day 3** | **Day 4** | **Day 5** |
| **Mains** |  |  |  |  |  |
| Pizza | 5 | 17 | 3 | 5 | 3 |
| Bread ^c^ | 18 | 13 | 14 | 19 | 11 |
| Pasta | 3 | 3 | 8 | 4 | 1 |
| Chips ^d^ | 4 | 11 | 8 | 5 | 6 |
| Other | 6 | 4 | 8 | 4 | 1 |
| Missing | 2 | 3 | 3 | 6 | 18 |
|  |  |  |  |  |  |
| **Dessert** |  |  |  |  |  |
| Fruit | 4 | 4 | 0 | 1 | 0 |
| Sugar-based ^e^ | 17 | 21 | 25 | 20 | 16 |
| None | 3 | 1 | 2 | 2 | 2 |
| Missing | 14 | 12 | 11 | 15 | 20 |
|  |  |  |  |  |  |
| **Drink** |  |  |  |  |  |
| Water | 12 | 14 | 13 | 8 | 7 |
| Other ^f^ | 12 | 12 | 10 | 13 | 10 |
| None | 2 | 0 | 3 | 4 | 1 |
| Missing | 12 | 12 | 12 | 13 | 20 |
|  |  |  |  |  |  |
| **Were fruit and vegetables present in meal?** | |  |  |  |  |
| No | 11 | 9 | 11 | 10 | 11 |
| Yes | 21 | 18 | 14 | 11 | 9 |
| Missing | 6 | 11 | 13 | 17 | 18 |
|  |  |  |  |  |  |
| **If you had more money would you have chosen the same thing?** | | | | |  |
| Yes | 15 | 10 | 12 | 8 | 9 |
| No | 10 | 11 | 9 | 7 | 4 |
| Missing | 13 | 17 | 17 | 23 | 25 |
|  |  |  |  |  |  |
| Yes | 12 | 5 | 9 | 4 | 5 |
| No | 9 | 13 | 9 | 8 | 7 |
| Missing | 17 | 20 | 20 | 26 | 26 |
|  |  |  |  |  |  |
| **Did the food you were able to buy at lunchtime fill you up?** | | | | |  |
| Fully full | 10 | 13 | 14 | 13 | 9 |
| Somewhat full | 15 | 8 | 11 | 6 | 5 |
| Not very full | 8 | 6 | 2 | 2 | 2 |
| Still very hungry | 2 | 3 | 3 | 1 | 1 |
| Missing | 3 | 8 | 8 | 16 | 21 |
| ^a^ The number of food items consumed within “mains” is independent to the number of food records, therefore the column total will exceed the 190 food records. For instance, pupils could consume both pizza and chips. The number of missing data refers to the number of pupils who did not complete a food record. ^b^ Meals which included paninis, baguettes, garlic bread, wraps, naan, loaf.  ^c^ Percentage of reported data based on number of food records across the week. This has been calculated as the total of 190 entries minus sum of missing data over 5 days. ^d^ This included chips, fries, wedges. ^e^ This included cakes, biscuits, baked goods and other desserts containing refined sugar.  ^f^ This included fruit juice, Radnor Fizz and other flavoured water. | | | | | |
