# Supplementary material for: “Will my fingerprint be enough?”: Secondary school students struggle to purchase a healthy, tasty and sustainable meal on the UK free school meal allowance
Source: Public Health Nutr. Author manuscript; Available in PMC 2025 Jan 28. (PMC7617354; doi:10.1017/S1368980024002593)
Supplement: Supplementary file 1 [file EMS202679-supplement-Supplementary_Table_1.pdf]

Supplementary Table 1. Frequency of citizen scientists' food choices and perceptions daily (n=38)

Item	Day 1	Day 2	Day 3	Day 4	Day 5
**Mains**					
Pizza	5	17	3	5	3
Bread [c]	18	13	14	19	11
Pasta	3	3	8	4	1
Chips [d]	4	11	8	5	6
Other	6	4	8	4	1
Missing	2	3	3	6	18
**Dessert**					
Fruit	4	4	0	1	0
Sugar-based [e]	17	21	25	20	16
None	3	1	2	2	2
Missing	14	12	11	15	20
**Drink**					
Water	12	14	13	8	7
Other [f]	12	12	10	13	10
None	2	0	3	4	1
Missing	12	12	12	13	20
**Were fruit and vegetables present in meal?**					
No	11	9	11	10	11
Yes	21	18	14	11	9
Missing	6	11	13	17	18
**If you had more money would you have chosen the same thing?**					
Yes	15	10	12	8	9
No	10	11	9	7	4
Missing	13	17	17	23	25
Yes	12	5	9	4	5
No	9	13	9	8	7
Missing	17	20	20	26	26
**Did the food you were able to buy at lunchtime fill you up?**					
Fully full	10	13	14	13	9
Somewhat full	15	8	11	6	5
Not very full	8	6	2	2	2
Still very hungry	2	3	3	1	1
Missing	3	8	8	16	21

[a] The number of food items consumed within "mains" is independent to the number of food records, therefore the column total will exceed the 190 food records. For instance, pupils could consume both pizza and chips. The number of missing data refers to the number of pupils who

did not complete a food record.
[b] Meals which included paninis, baguettes, garlic bread, wraps, naan, loaf.
[c] Percentage of reported data based on number of food records across the week. This has been calculated as the total of 190 entries minus sum of missing data over 5 days.
[d] This included chips, fries, wedges.
[e] This included cakes, biscuits, baked goods and other desserts containing refined sugar.
[f] This included fruit juice, Radnor Fizz and other flavoured water.
